# Predicting Job Satisfaction in Military Organizations: Unpacking the Relationship Between Emotional Intelligence, Teamwork Communication, and Job Attitudes in Spanish Military Cadets

**DOI:** 10.3389/fpsyg.2020.00875

**Published:** 2020-05-07

**Authors:** Inmaculada Valor-Segura, Ginés Navarro-Carrillo, Natalio Extremera, Luis M. Lozano, Carlos García-Guiu, María Isabel Roldán-Bravo, Antonia Ruiz-Moreno

**Affiliations:** ^1^Department of Social Psychology, Faculty of Psychology, Mind, Brain and Behavior Research Center, University of Granada, Granada, Spain; ^2^Department of Psychology, University of Jaén, Jaén, Spain; ^3^Department of Social Psychology, Faculty of Psychology, University of Málaga, Málaga, Spain; ^4^Department of Research Methods in Behavioral Sciences, Faculty of Psychology, Mind, Brain and Behavior Research Center, University of Granada, Granada, Spain; ^5^Spanish General Military Academy, Zaragoza, Spain; ^6^Centro Mixto University of Granada-Madoc, Granada, Spain; ^7^Department of Business Administration, University of Jaén, Jaén, Spain; ^8^Department of Business Organization, Faculty of Economics and Business Management, University of Granada, Granada, Spain

**Keywords:** emotional intelligence, job satisfaction, communication, military context, proactive personality, resilience

## Abstract

Although prior research has extensively examined the association of emotional intelligence (EI) with various job attitudes (e.g., job satisfaction), empirical and systematic investigation of this link within military institutions has captured considerably less attention. The present research analyzed the relationship between EI, teamwork communication, and job satisfaction among Spanish military cadets. We tested the potential unique contribution of EI to job satisfaction over and above demographics (i.e., gender and age), proactive personality, and resilience. Moreover, we also examined whether EI was indirectly linked to job satisfaction via its relationship with teamwork communication. A sample of 363 cadet officers of the Spanish General Military Academy completed questionnaires assessing EI, teamwork communication, proactive personality, resilience, and job satisfaction. Hierarchical regression analysis revealed that EI exhibited incremental variance (Δ*R*^2^ = 5.2%) in predicting job satisfaction (*B* = 0.539, 95% CI [0.306,0.771]) even after accounting for demographics, proactive personality, and resilience. Additionally, mediation analysis showed that the association of EI with job satisfaction was partially driven by enhanced teamwork communication. This research provides empirical evidence suggesting a pathway (i.e., effective teamwork communication) through which EI could help military cadets to experience higher job satisfaction. Implications for future academic programs including EI and teamwork communication to promote positive job attitudes among military personnel are discussed.

## Introduction

Emotional intelligence (EI) has been conceptualized as an individual difference dimension that encompasses a set of abilities concerned with the processing of emotion-relevant information. According to Mayer and Salovey’s (1997) theoretical approach, EI could be defined as “the ability to perceive emotions, to access and generate emotions so as to assist thought, to understand emotions and emotional knowledge, and to reflectively regulate emotions so as to promote emotional and intellectual growth” (Mayer and Salovey, 1997, p. 5). In the last two decades, a significant body of research has documented the predictive validity of EI across a wide array of psychological domains. For instance, EI has been found to predict a set of health-related dimensions and behaviors (Martins et al., 2010; Fernández-Abascal and Martín-Díaz, 2015), subjective well-being (Sánchez-Álvarez et al., 2016), cognitive and affective academic engagement (Maguire et al., 2017), career decision making (Farnia et al., 2018), or social sharing motives (Bucich and MacCann, 2019), among others.

A promising avenue for future research is related to the examination of psychological processes involved between individuals’ EI and critical work-related outcomes (e.g., O’Boyle et al., 2011; Miao et al., 2017a). Accordingly, given that people high in EI are more prone to successfully evaluating and regulating their emotional states (Peña-Sarrionandia et al., 2015), thereby deploying more appropriate strategies for coping with adverse circumstances, employees’ EI is argued to play a fundamental role in the maintenance and development of positive individual and organizational outcomes. Indeed, higher EI has been consistently and positively associated with positive work outcomes, such as job satisfaction (Meisler and Vigoda-Gadot, 2014; Rezvani et al., 2016; Extremera et al., 2018), trust and project success (Rezvani et al., 2016), psychological ownership of the job (Kaur et al., 2013), or organizational commitment (Naderi Anari, 2012). Although the aforementioned studies substantially strengthen the idea that EI may promote desirable work outcomes, it is important to ascertain whether the effects of EI remained significant once established, well-known personality or work-related constructs are controlled in the analytical models. However, there is growing valuable research trying to demonstrate incremental validity of EI in predicting work attitudes and behavior. For example, Carmeli (2003) found that EI predicted a set of work outcomes (i.e., job performance, withdrawal intention, altruistic behavior, career commitment, affective commitment, work-family conflict, and job satisfaction) even after accounting for age, income, organizational size, and tenure in an organization. Further studies have also shown that EI uniquely predicted burnout levels (beyond demographic and work-related factors; Platsidou, 2010) and entrepreneurial self-efficacy (above and beyond demographics and personality traits; Mortan et al., 2014).

The potential unique connection of EI with various positive work outcomes has gained increasing attention from meta-analytic research in last years. In this vein, recent meta-analytic findings have indicated that EI exhibited incremental validity in the prediction of job satisfaction, organizational commitment, and turnover intentions above and beyond the Big Five personality factors (Miao et al., 2017a), thus suggesting the unique contribution of EI to these work attitudes. Moreover, Miao et al. (2017a) also explored potential theoretically related psychological mechanisms that might act as mediator variables in the association between EI and job satisfaction. In particular, their study demonstrated that both state affect and job performance mediated the EI–job satisfaction relationship. Similarly, in another meta-analysis aimed at exploring the effects of EI on job satisfaction it was also found that employees with higher EI reported greater job satisfaction (Miao et al., 2017b); notably, this relationship existed independently of numerous relevant employees’ characteristics (i.e., gender, age, and tenure). In additional support of the unique contribution of EI to work outcomes, another recent meta-analysis of EI provided empirical evidence showing that EI has incremental validity in predicting both organizational citizenship behavior and counterproductive work behavior after controlling for classical variables, such as personality dimensions or general self-efficacy (Miao et al., 2017c).

Overall, the data above suggest that there is increasing convergence on the idea that EI represents a central individual characteristic for uniquely enhancing positive individual and work-related outcomes in organizational life. However, even though the positive effects of employees’ EI on job satisfaction across organizational settings have received accumulating evidence, empirical and systematic investigation of such a relationship within military organizations has captured considerably less scholarly attention (Shi et al., 2015). Currently, military organizations are mainly hierarchical and large institutions where orders have a great impact on subordinate members (Ramthun and Matkin, 2014). Operating in complex socio-cultural (military forces are integrated in local populations), globalized (mass media and social platforms could trigger far-reaching incidents), and rapidly changing (a wide range of operations are performed, from maintaining security to fighting) environments represent a major challenge for the military culture and organization. Therefore, these institutions should ineluctably provide these cadet officers’ abilities to perceive and regulate their emotions within the particularly complex military environment. For example, in a large cohort of North American military academy cadets, research confirmed the importance of cognitive and personality variables that could contribute to military competences and performance (Bartone et al., 2002). Nevertheless, EI was not specifically measured in that research. Thus, these authors suggest the need to explore additional factors that may influence military performance beyond reasoning, personality, and social skills. In this military context, an increasing number of authors uphold the importance of acknowledging the pivotal role of EI as a resource to be considered for selecting military leaders and training them to be more self-aware, flexible, adaptive, and transformative (Abrahams, 2007; Sewell, 2009; Deveci, 2016). Moreover, this approach is reinforced because the attributes and competences of Army officers have showed significant associations with EI (Taylor-Clark, 2015; Koh and O’Higgins, 2018). Although some studies have highlighted in a generic manner the importance of cognitive, emotional, and social-related factors within educational and training military institutions (e.g., Devine, 2012), as well as robust connections between EI and positive job attitudes (Lam and O’Higgins, 2012), the examination of the implications of EI on job attitudes and the potential underlying psychological factors within educational military environments is still limited. It is therefore imperative to increase our empirical knowledge on the role of EI in this context and subsequently to understand the mediating mechanisms through which EI training programs are effective to foster positive job outcomes, and ways to widely disseminate research-based intervention into military schools.

Through this research, we intend to extend prior research findings on EI by elucidating the specific role of EI in job satisfaction—one of the central variables in the organizational field (Judge and Kammeyer-Mueller, 2012)—among military forces, while also controlling for the potential influence of further conceptually related personality constructs, such as individuals’ levels of proactive personality and resilience. Although recent research has found that both proactive personality and resilience are positively correlated with EI and job satisfaction (Jafri et al., 2016; Di Fabio and Saklofske, 2018; Kašpárková et al., 2018; Kuo et al., 2019), no studies have analyzed whether EI predicts job satisfaction even after accounting for proactive personality and resilience.

Additionally, considering that the underlying psychological mechanisms of the EI–job satisfaction association are barely known (Extremera et al., 2018), we also explored whether teamwork communication—a basic teamwork competence (Aguado et al., 2014)—would act as a plausible mediating psychological variable. Previous research has suggested that high EI could represent a prerequisite to developing adequate and effective interpersonal communication skills (Goleman, 1995). Nonetheless, the role of teamwork communication in the relationship between EI and job satisfaction has not been elucidated so far. In this regard, prior indirect evidence allows us to infer that communication competence could exert an explanatory role. For instance, EI has been proven to be positively correlated with perceptions of constructive communication patterns (Smith et al., 2008). Furthermore, the adequate communication levels within the working group constitute a factor with resultant effectiveness and cooperation (George, 2002; Jordan et al., 2002; Pandey and Karve, 2018). Indeed, effective communication at work has been found to correlate with higher job satisfaction (Kim, 2002), thereby supporting the beneficial impact of communication on positive job attitudes. Because EI is deemed a key factor in various types of communication, potentially leading to optimal work-related outcomes (e.g., Sinha and Sinha, 2007), one might expect that EI positively affects job satisfaction by enhancing teamwork communication. Teamwork communication competence can be understood as the ability to identify and use decentralized networks to boost communication, as well as to use communication following both open and supportive techniques, or to adequately capture the non-verbal messages of other individuals (Stevens and Campion, 1999). Addressing interpersonal communication skills within the framework of a military teamwork is key as the team represents the major formation unity, and this is the scenario where the main social activities and collaborative work tasks are developed (Bass et al., 2003; Griffith, 2007). Indeed, instructional and training practices take place in these formation unities which are based upon communication-related aspects between the team components, providing insight on psychological dynamics and outcomes related to EI (Stubbs and Wolff, 2008). Overall, examining the role of teamwork communication in military units might help improve professional readiness and adopt more updated and effective military training practices and programs to promote positive work outcomes (Oden et al., 2015; Deveci, 2016).

## The Current Study

**FIGURE 1:**
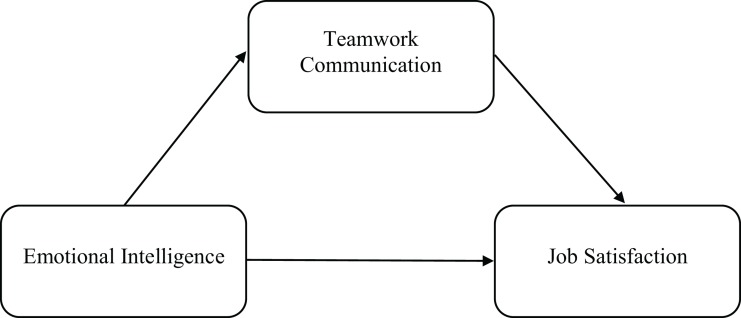
Proposed model of the role of teamwork communication in explaining the emotional intelligence–job satisfaction relationship.

This research is principally aimed at testing the predictive ability of EI on job satisfaction among a large sample of cadet officers. Additionally, we also examined the incremental validity of EI above and beyond partially conceptual overlapping factors (i.e., proactive personality and resilience), as well as the putative mediating role of work-team communication competence in the EI–job satisfaction relationship (see Figure 1), thus complementing prior research that analyzed other mediating factors (e.g., Miao et al., 2017a; Extremera et al., 2018). Overall, proceeding on the basis of the abovementioned considerations, the following hypotheses have been made:

*Hypothesis 1*: Cadet officers’ EI will predict increased job satisfaction.
*Hypothesis 2*: Cadet officers’ EI will exhibit incremental validity in job satisfaction above and beyond the effects of proactive personality and resilience.
*Hypothesis 3*: Teamwork communication competence will act as a mediator in the relationship between cadet officers’ EI and job satisfaction.

## Materials and Methods

### Sample

Cadet officers of the Spanish General Military Academy, located in Zaragoza, Spain, were invited to participate in this research. A total of 363 individuals completed a comprehensive survey including five separate questionnaires. The sample comprised 343 male and 20 female military cadets between 20 and 40 years of age (*M* = 23.48, SD = 3.99).

## Measures

### Emotional Intelligence

We used the Wong Law Emotional Intelligence Scale (WLEIS-S; Wong and Law, 2002). This instrument consists of 16 items measuring four aspects of EI: Self-Emotion Appraisal, SEA, Others’ Emotion Appraisal, OEA, Use of Emotion, UOE, and Regulation of Emotion, ROE. However, as in prior studies, we used the overall score in our analyses as we were interested in the global EI score (Law et al., 2004; Mérida-López et al., 2019). Items (e.g., “I am quite capable of controlling my own emotions” or “I always encourage myself to try my best”) were rated using a 7-point Likert scale ranging from 1 (*strongly disagree*) to 7 (*strongly agree*). Higher scores in this measure reflect higher levels of EI (α = 0.86). We used the well-validated Spanish version (Extremera et al., 2019).

### Teamwork Communication

We used the communication measure composed of eight items of the Teamwork Knowledge, Skill, Ability Test proposed by Stevens and Campion (1994, 1999) and adapted to the Spanish population (Aguado et al., 2014; e.g., “I make an effort to talk about less important things with my peers for the sake of team spirit and better internal communication”). The answer format is a Likert-type scale with five options ranging from 0 (*never*) to 4 (*always*); high scores indicate effective communication for teamwork. Cronbach’s alpha reliability on the present sample was 0.76.

### Proactive Personality

We administered the shortened version of the Proactive Personality Scale proposed by Seibert et al. (2001) and validated according to Bateman and Crant’s (1993) work. The short version consists of 10 items (e.g., “I am constantly on the lookout for new ways to improve my life”) measuring individual differences in the inclination to take action and change the environment. Items were evaluated using a 7-point Likert scale ranging from 1 (*strongly disagree*) to 7 (*strongly agree*). Higher scores indicate higher levels of proactive personality. Cronbach’s alpha reliability was 0.86.

### Resilience

We used the 5-item measure developed by Hardy et al. (2010) to evaluate resilience. It was operationalized as the ability to maintain confidence in the face of misadventures and dissatisfaction experiences (e.g., “Bounce back from performing poorly and succeed”). The response format was a 5-point Likert scale anchored at 1 (*low*) to 5 (*high*). Higher scores reflect greater resilience. Cronbach’s alpha reliability was 0.81.

### Job Satisfaction

To assess job satisfaction, we administered the job satisfaction scale proposed by Judge et al. (1998); this measure was based on Brayfield and Rothe’s (1951) overall job satisfaction scale (e.g., “Most days I am enthusiastic about my work”). The responses to its 5 items were measured using a 7-point scale ranging from 1 (*strongly disagree*) to 7 (*strongly agree*). Higher scores in this measure indicate increased job satisfaction. In this study, the Cronbach’s alpha coefficient was 0.84.

## Procedure

A number of previously trained evaluators requested military cadets’ volunteers to participate in the Spanish General Military Academy. The evaluators explained to participants how the collected information will be used, ensuring that they could abandon their participation in the study at any time and without any consequences. After participants were informed about the estimated duration of their collaboration (about 25 min), confidentiality, and anonymity regarding their answers, they proceeded to complete the questionnaire booklet individually in Spanish General Military Academy classrooms while supervised by the aforementioned evaluators. Volunteers did not receive any type of academic or financial compensation in exchange for their participation. All respondents gave their informed consent for inclusion before they participated in the study. The study was conducted in accordance with the Declaration of Helsinki and the protocol was approved by the Ethics Committee of CEMIX-UGR-MADOC (Ref. 22/18).

## Statistical Analysis

First, basic descriptive statistics (i.e., means and standard deviations) and bivariate correlations for all measures were computed. Then, we performed a hierarchical regression analysis to determine the possible unique predictive contribution of EI to job satisfaction. As a preliminary check, we calculated variance inflation factors (VIFs) for each independent questionnaire variable. Collinearity statistics obtained for our sample showed acceptable values (VIFs < 1.57; Akinwande et al., 2015). Lastly, we further computed a simple mediation analysis to explore whether the EI–job satisfaction relationship among cadet officers could be explained—at least, partially—by teamwork communication competence levels. The analyses were conducted using Statistical Package for the Social Sciences (SPSS version 23.0; SPSS Inc., Chicago, IL, United States).

## Results

### Descriptive Statistics and Correlation Analysis

Descriptive statistics (i.e., means and standard deviations) and product–moment correlation coefficients for all key variables in the study are given in Table 1. Aligning with our main expectations, military cadets’ EI showed positive correlations with the rest of the variables (Table 1). Thus, greater EI was significantly associated with elevated teamwork communication competence, proactive personality, resilience, and job satisfaction.

**TABLE 1:** Means, standard deviations, and correlations for EI, communication competence, proactive personality, resilience and job satisfaction.

**Variable**	***M***	**SD**	**1**	**2**	**3**	**4**	**5**
1. Emotional intelligence	5.49	0.63	*–*	*–*	*–*	*–*	*–*
2. Communication	2.75	0.55	0.45***	*–*	*–*	*–*	*–*
3. Proactive personality	5.46	0.66	0.51***	0.36***	*–*	*–*	*–*
4. Resilience	3.96	0.53	0.51***	0.37***	0.46***	*–*	*–*
5. Job satisfaction	4.85	1.20	0.36***	0.26***	0.29***	0.26***	*–*

***p < 0.001.

### The Unique Contribution of EI to Job Satisfaction

To verify Hypotheses 1 and 2, we performed a multiple hierarchical regression analysis. In the first step of the regression equation, demographics (i.e., sex and age) were entered (method: enter). Proactive personality and resilience were included in the second step (method: enter). Finally, we incorporated EI in the third step (method: enter) to calculate its added value in explaining variance in job satisfaction and ascertain its unique predictive contribution to this criterion above and beyond demographics (i.e., gender and age), proactive personality, and resilience. The results of the regression analysis predicting cadet officers’ job satisfaction are given in Table 2.

**TABLE 2:** Summary of the hierarchical regression analysis with job satisfaction as the criterion variable.

**Predictor**	**Job satisfaction**			
	**B**	**CI (95%)**	***p***	***R*^2^**
**Step 1**				0.002
Gender	−0.067	[−0.604, 0.471]	0.808	
Age	−0.014	[−0.052, 0.025]	487	
**Step 2**				0.101
Gender	−0.044	[−0.557, 0.469]	0.866	
Age	−0.013	[−0.050, 0.025]	0.510	
Proactive personality	0.369	[0.160, 0.578]	0.001	
Resilience	0.368	[0.106, 0.630]	0.006	
**Step 3**				0.153
Gender	0.003	[−0.496, 0.503]	0.990	
Age	−0.023	[−0.060, 0.014]	0.222	
Proactive personality	0.180	[−0.039, 0.399]	0.107	
Resilience	0.160	[−0.111, 0.430]	0.246	
Emotional intelligence	0.539	[0.306, 0.771]	<0.001	

*Unstandardized regression coefficients are reported. CI, confidence interval.*

Neither gender nor age significantly contributed to the prediction of job satisfaction, with an amount of criterion variance explained of 0.2%, *F*(2,341) = 0.309, *p* = 0.735. Conversely, the model including proactive personality and resilience, which were added at the second step of the regression analysis, was significant, *F*(4,339) = 9.523, *p* < 0.001. As illustrated in Table 2, both proactive personality and resilience emerged as significant predictors of higher levels of job satisfaction. These personality-related factors explained an additional 10% of the variance in job satisfaction. The inclusion of EI in the third step of the regression equation accounted for incremental criterion variance (5.2%), and the regression model remained significant, *F*(5,338) = 12.231, *p* < 0.001. As Table 2 shows, the regression coefficient for EI was significantly positive, *t*(343) = 4.564, *p* < 0.001, indicating that military cadets’ EI levels uniquely predicted greater job satisfaction even after controlling for demographics (i.e., gender and age) and personality variables (i.e., proactive personality and resilience). Hence, Hypotheses 1 and 2 are supported.

### The Mediating Role of Teamwork Communication

We used Hayes’ PROCESS macro for SPSS (Model 4; Hayes, 2013) to test Hypothesis 3, namely to determine the potential indirect effect of EI on job satisfaction via the mediator variable (i.e., communication). None of the covariate (age, gender, proactive personality, and resilience) effects were significant (all *ps* > 0.13). The only exception was the effect of proactive personality on teamwork communication (*b* = 0.173, SE = 0.059, *p* = 0.003). The results showed that military cadets with greater scores on EI showed higher communication competence (*b* = 0.240, SE = 0.059, *p* < 0.001), which in turn was related to increased job satisfaction (*b* = 0.391, SE = 0.151, *p* = 0.010). We calculated 95% bias-corrected confidence intervals (CIs) for the point estimate on the basis of 5,000 bootstrap samples. Taking into account Hayes’ (2013) indications, the indirect effect is considered statistically significant when the 0 value is not included in its CI. Given that 0 is outside the CI [0.015, 0.204] of the indirect effect of cadet officers’ EI on job satisfaction via communication competence (*b* = 0.094) (Table 3), the results confirmed that communication competence mediated the EI–job satisfaction relationship; hence, military cadets’ EI was indirectly linked to job satisfaction through its association with teamwork communication. After controlling for the effect of communication (i.e., mediator variable), the direct effect of EI on job satisfaction among cadet officers remained significant (*b* = 0.435, SE = 0.140, *p* = 0.002, 95% CI [0.160, 0.711]), thus indicating the existence of a partial mediation. Thus, Hypothesis 3 is supported.

**TABLE 3:** Summary of mediation analysis.

	**Outcome: Communication**				**Outcome: Job satisfaction**			
**Predictors**	**Coeff.**	**SE**	**LLCI**	**ULCI**	**Coeff.**	**SE**	**LLCI**	**ULCI**
Constant	–0.021	0.373	–0.755	0.713	1.548	0.855	–0.137	3.233
Co: Gender	0.094	0.145	–0.192	0.380	0.277	0.334	–0.380	0.935
Co: Age	–0.001	0.010	–0.019	0.018	–0.033	0.022	–0.076	0.010
Co: Proactive personality	0.173**	0.059	0.057	0.288	–0.004	0.137	–0.274	0.266
Co: Resilience	0.128	0.070	–0.011	0.266	0.177	0.163	–0.143	0.498
Me: Communication	–	–	–	–	0.391**	0.151	0.094	0.688
X: EI	0.240***	0.059	0.124	0.356	0.435**	0.140	0.160	0.711
*R*^2^	0.261***	–	–	–	0.164***	–	–	–

**Indirect effect**		**Boot SE**			**Boot LLCI**		**Boot ULCI**	

0.094		0.048			0.015		0.204	

Unstandardized regression coefficients are reported. Bootstrap simple size: 5,000. LLCI = lower level of the 95% bootstrap percentile confidence interval; ULCI = upper level of the 95% bootstrap percentile confidence interval. The indirect effect is significant where the confidence intervals does not contain zero. **p < 0.01; ***p < 0.001.

## Discussion

In the present research, we examined the predictive and incremental validity of EI on job satisfaction above and beyond the effects attributable to common demographic characteristics (i.e., gender and age), proactive personality, and resilience in Spanish military cadets; furthermore, this is also the first study to explore the mediating role of teamwork communication competence in the relationship between EI and job satisfaction in the military context.

Our regression findings supported Hypotheses 1 and 2. In line with prior meta-analytic research, EI has demonstrated its ability to predict some significant variance over levels of job satisfaction (Miao et al., 2017a). Besides, strong predictors of job satisfaction such as proactive personality and resilience have not been controlled for in previous research. Given the functional similarities among EI, proactivity, and resilience in predicting positive attitudes at workplace, we were also interested in verifying the contribution of EI to job satisfaction beyond the influence of cadet officers’ other traits characteristics. Our results showed that EI explained a significant proportion of variance in job satisfaction above and beyond the effects of demographic variables, proactive personality, and resilience. These findings provide a stringent test of the practical importance of EI as an explanatory factor of job satisfaction in military organizations over theoretically and empirically robust predictors. Moreover, in our study EI explained an additional 5% of the variance of levels of job satisfaction. While this explained incremental variance was not excessively large, incremental values like these should be considered a reasonable contribution when other variables are controlled (Haynes and Lench, 2003; Hunsley and Meyer, 2003). In sum, independent of the influence of other well-known dispositional traits and demographics variables, our set of results has indicated that EI also plays a significant role in how cadet officers could develop and maintain positive job attitudes, suggesting that intervention programs focused on cultivating EI might have beneficial effects in the development and maintenance of job satisfaction among military members (Miao et al., 2017a; Mattingly and Kraiger, 2019).

On the other hand, the mediation analysis also demonstrated a partial mediation effect of teamwork communication competence in the relationship between EI and job satisfaction. Extending past research on mediating mechanisms in the link between EI and job satisfaction (Extremera et al., 2018), our study found that emotionally intelligent cadet officers reported higher effective communication for teamwork, which in turn was related to higher job satisfaction. Emotionally intelligent workers are thought to show not only higher interpersonal skills but also greater levels of effective communication for teamwork (Hendon et al., 2017). According to prior research (Lopes et al., 2004; Lopes et al., 2011), military cadets with higher EI might communicate more effectively because they are able to perceive, understand, and implement strategies to infer other people’s intentions from their affective signs, use others’ emotions as guides for their behavior, or influence people’s motivation and use of effective skills to maintain successful relations that might result in greater job satisfaction. Military activity has been found to be associated with an array of workplace stressors that could affect the development of job attitudes (Laurence and Matthews, 2012). EI is proposed as a psychological resource that might contribute to developing positive job attitudes and behaviors and, specifically, job satisfaction (Miao et al., 2017a). According to our findings, one potential mechanism by which military cadets’ EI may help to explain higher job satisfaction is through using and developing effective teamwork communication.

### Limitations and Future Directions

Some limitations of our study are to be mentioned. First, our results are based on self-reported data and we used a cross-sectional design. Therefore, this methodology makes it impossible to determine the direction of association between variables. Further studies should include other complementary measurement approaches (e.g., interviews and situational judgment tests) and use longitudinal designs that provide further insights to the causal relationships between EI, teamwork communication, and positive job attitudes among cadet officers. Although one strength of this study was the examination of the relationship of EI and job satisfaction in a non-commercial organization (i.e., Spanish General Military Academy), one limitation is that the military academy is an officer training and education context, and positive attitudes in this context might not be strongly related with their positions in army units after graduation.

Despite these limitations, our study provides insights into how EI, teamwork communication, and positive attitudes in military organizations are connected, and how the relationship between EI and job satisfaction is independent of other well-known dispositional factors. These findings might help to design future intervention programs aimed at increasing EI and teamwork communication skills among future career officers. EI is a psychological construct that might be predictive of several positive functioning outcomes and might foster adaptive (and mitigate maladaptive) personal and organizational outcomes for active duty members and their families (Zeidner et al., 2012; Bowles et al., 2015). In military operations, the success of missions frequently depends on the capacity of military leaders to understand and manage the emotions of the team and on the effective team communication among members in challenging and dangerous environments (Campbell, 2012). Current conflicts involving non-state actors require military leaders to be adaptive to shifting roles where EI favors interpersonal relationships, regulating stress, and focusing attention to optimize decision making (Jeppesen, 2017). Further academic programs of cadet leadership instruction should focus on developing EI and enable participants to acquire skills and tools to promote effective team interaction strategies for smooth communication and positive interpersonal relationships. The development of these abilities might help officers to effectively work with others in groups and teams. Notwithstanding this, it is worth mentioning that, given that our research has directly assessed respondents’ perceptions of interpersonal communication-related aspects within a team, thus focusing on the individual level, further empirical research within the military context is needed to extend and complement our findings by incorporating team-related approaches. Overall, military institutions could benefit from implementing programs to facilitate and foster emotional abilities and effective teamwork communication to promote both personal and job attitudes (Mencl et al., 2016).

### Conclusion

In conclusion, the setting for cadet officers typically involves collaborative and interdisciplinary teamwork tasks. However, working collaboratively requires several personal skills to allow different military academy cadets to effectively synchronize, work together cooperatively to solve conflict problems, and contribute as members of inter-professional teams to provide successful performance outcomes and experience higher positive attitudes in their daily military tasks. Increasing their abilities to understand and to manage their emotions may be positively related to teamwork communication and, therefore, positively linked to job attitudes. Extending previous findings on incremental validity of EI and underlying mechanisms between EI and job attitudes (Miao et al., 2017a), our current findings provide empirical support of (a) the incremental role of EI in job satisfaction beyond further dispositional factors and (b) the mediating role of teamwork communication in the EI–job satisfaction association in the military context. In short, teamwork communication skills appear to be a partial mediator in the association between emotional skills and job satisfaction in Spanish cadet officers. Therefore, team communication skills and emotional abilities may represent promising targets for academic training programs aimed at increasing job satisfaction among military cadets. If our findings are replicated, then these emotional and teamwork skills might become an integral part of a training module for the development of strategies for improving positive attitudes at work for future career officers.

## Data Availability Statement

The datasets generated for this study are available on request to the corresponding author.

## Ethics Statement

The studies involving human participants were reviewed and approved by the Ethics Committee of CEMIX-UGR-MADOC (Ref. 22/18). The patients/participants provided their written informed consent to participate in this study.

## Author Contributions

IV-S, CG-G, and AR-M: project design, administration and funding acquisition. LL: formal analysis and data curation. GN-C, IV-S, NE, and CG-G: writing–original draft preparation. All authors: writing–review and editing.

## Conflict of Interest

The authors declare that the research was conducted in the absence of any commercial or financial relationships that could be construed as a potential conflict of interest.
